# In vivo deuterium magnetic resonance imaging of xenografted tumors following systemic administration of deuterated water

**DOI:** 10.1038/s41598-023-41163-9

**Published:** 2023-09-07

**Authors:** Jeffrey R. Brender, Julian C. Assmann, Don E. Farthing, Keita Saito, Shun Kishimoto, Kathrynne A. Warrick, Natella Maglakelidze, Terri L. Larus, Hellmut Merkle, Ronald E. Gress, Murali C. Krishna, Nataliya P. Buxbaum

**Affiliations:** 1 Radiation Biology Branch, Center for Cancer Research, National Cancer Institute, National Institutes of Health, Bethesda, MD USA; 2 Experimental Transplantation and Immunotherapy Branch, National Cancer Institute, National Institutes of Health, Bethesda, MD USA; 3 Center for Immuno-Oncology, Center for Cancer Research, National Cancer Institute, National Institutes of Health, Bethesda, MD USA; 4 Laboratory for Functional and Molecular Imaging, National Institute of Neurological Disorders and Stroke, National Institutes of Health, Bethesda, MD USA; 5 Pediatric Oncology, Roswell Park Comprehensive Cancer Center, Buffalo, NY USA

**Keywords:** Cancer imaging, Imaging techniques

## Abstract

In vivo deuterated water (^2^H_2_O) labeling leads to deuterium (^2^H) incorporation into biomolecules of proliferating cells and provides the basis for its use in cell kinetics research. We hypothesized that rapidly proliferating cancer cells would become preferentially labeled with ^2^H and, therefore, could be visualized by deuterium magnetic resonance imaging (dMRI) following a brief period of in vivo systemic ^2^H_2_O administration. We initiated systemic ^2^H_2_O administration in two xenograft mouse models harboring either human colorectal, HT-29, or pancreatic, MiaPaCa-2, tumors and ^2^H_2_O level of ~ 8% in total body water (TBW). Three schemas of ^2^H_2_O administration were tested: (1) starting at tumor seeding and continuing for 7 days of in vivo growth with imaging on day 7, (2) starting at tumor seeding and continuing for 14 days of in vivo growth with imaging on day 14, and (3) initiation of labeling following a week of in vivo tumor growth and continuing until imaging was performed on day 14. Deuterium chemical shift imaging of the tumor bearing limb and contralateral control was performed on either day 7 of 14 after tumor seeding, as described. After 14 days of in vivo tumor growth and 7 days of systemic labeling with ^2^H_2_O, a clear deuterium contrast was demonstrated between the xenografts and normal tissue. Labeling in the second week after tumor implantation afforded the highest contrast between neoplastic and healthy tissue in both models. Systemic labeling with ^2^H_2_O can be used to create imaging contrast between tumor and healthy issue, providing a non-radioactive method for in vivo cancer imaging.

## Introduction

Imaging is an essential tool for cancer diagnosis, staging, and surveillance. Positron emission tomography (PET) in combination with either computer tomography (CT) or magnetic resonance imaging (MRI) is commonly used in this setting. These imaging techniques provide clinically relevant information that can guide therapeutic decisions^1,2^. Radioactive tracers, such as ^18^fluorodeoxyglucose (FDG), exploit the high glycolytic activity of cancer cells to reveal tumors that are not readily detectable by anatomical imaging alone^3^. Such detection can result in clinically meaningful outcomes for patients, guiding surgical or medical interventions, including sensitive detection of cancer recurrence or metastasis. However, patients are exposed to ionizing radiation through PET-CT, which increases the risk for secondary malignancies, especially in children and adolescents^4–6^. While exposures from a single scan are relatively small, they are not insignificant and are estimated to be around 4–8 times higher than the average annual background radiation exposure^7,8^. Furthermore, serial imaging is often necessary to assess therapeutic responses and to monitor for disease recurrence, which is associated with additional radiation exposures^6,9^. In recent years, the rising use of PET-CT scans and other medical procedures that require the use of radiation has led to a 33% increase in medical procedure-related radiation exposure in the US population^10^.

MRI is a clinically useful cancer imaging method that does not involve ionizing radiation and can be used repeatedly and safely to evaluate treatment responses. From a research perspective, spectroscopic MRI approaches can give quantitative measurements of metabolism beyond intracellular uptake, which may provide greater insight into cancer biology than PET^11^. To trace the activity of specific enzymes, hyperpolarized ^13^C-MRI was developed. It uses dynamic nuclear polarization to facilitate the detection of ^13^C-labeled substrates (e.g., ^13^C-pyruvate) and their downstream metabolites^11,12^. Unfortunately, this method has been technically challenging to implement in clinical settings. Deuterium-labeled isotopes offer an alternative for metabolic imaging in vivo^13,14^. For example, deuterium metabolic imaging (DMI) was used to map differential metabolism of glucose within aggressive glioblastoma multiforme lesions compared to surrounding brain parenchyma^15^. However, glucose-based imaging techniques may have limited utility in certain tumor types, e.g., neuroendocrine, liver, lung, and prostate malignancies^16–18^. These limitations are at least partly due to the dependency of intratumoral glucose metabolism on the level of tissue perfusion and the physiologically high baseline rates of glycolysis in certain healthy tissues. To sustain high rates of proliferation malignant cells must generate new cellular biomass, while glucose is not the dominant source of carbon for new cell generation in tumors^19^.

Thus, we sought to develop an alternative cancer imaging approach based on our work with deuterated water labeling of rapidly proliferating cells^20^. During biosynthesis, (deuterated) water can be used as a substrate in multiple pathways, leading to the formation of stable carbon-deuterium bonds, thus allowing in vivo labeling of proliferating cells via an oral administration of deuterated water^21–23^. Deuterated water (^2^H_2_O) has long been used to study in vivo cell cycle kinetics in clinical and animal research^20,24–26^. In these studies, cells of interest are extracted from tissues or blood following systemic ^2^H_2_O intake, and deuterium incorporation into newly synthesized DNA strands is quantified by mass spectrometry to estimate cell proliferation rates. Similarly, systemic deuterated water labeling has also been used to quantitatively measure in vivo rates of carbohydrate, lipid, and protein metabolism^14,26–28^. We recently applied deuterium MRI (dMRI) to visualize target organs of graft-versus-host disease infiltrated by alloreactive T cells, which share certain features with tumor cells, i.e. rapid proliferation^20^ and altered metabolism^29^.

Spectroscopic^30,31^ and gravimetric studies^32,33^ indicate that carcinomas and sarcomas typically have a higher water content than normal tissue of the same ontogeny, a hallmark characteristic of rapidly growing cells^33,34^. We hypothesized that malignant cells that are highly proliferative will be differentially labeled with deuterium through systemic deuterated water administration leading to higher dMRI contrast compared to healthy tissues. To test this hypothesis, we used two xenograft models and found that systemic ^2^H_2_O labeling during the phase of rapid in vivo tumor growth, i.e., 1 week after initial seeding, led to a higher dMRI contrast in the tumor tissue compared to surrounding muscle, reaching a maximal point within a week. This suggests that deuterated water may serve as a contrast in distinguishing malignant from normal tissues, in addition to its incorporation into downstream metabolites.

## Methods

All methods are reported in accordance with ARRIVE guidelines (https://arriveguidelines.org).

### Mice

All experiments and procedures involving mice were approved by and carried out in accordance with the National Cancer Institute (NCI) Institutional Animal Use and Care Committee (IACUC) guidelines. Female athymic nude (Foxn1^nu^) mice aged 10–15 weeks were supplied by the Frederick Cancer Research Center (Frederick, MD, USA). Mice were kept under a 12 h/12 h light–dark cycle with ad libitum access to food and water.

### Cells

The human colorectal adenocarcinoma cell line HT-29 was purchased from ATCC (HTB-38), and the identity was confirmed using a panel of microsatellite markers (IDEXX Laboratories). HT-29 cells were cultured in RPMI 1640 supplemented with 10% fetal bovine serum, 100 U/ml penicillin, 100 µg/ml streptomycin and incubated at 5% CO_2_ and 37 °C. On the day of tumor injection, HT-29 cells were spun down at 1000 rpm for 5 min, resuspended in PBS and 1 × 10^6^ cultured cells were injected subcutaneously into the proximal hind limb of the mouse as previously published^35^. The contralateral hind limb did not receive a tumor cell injection and served as an intra-individual control. Similarly, the human pancreatic cancer cell line MiaPaCa-2 (CRL-1420) was cultured as described above and 3 × 10^6^ cells were injected subcutaneously into one hind limb while the contralateral limb was not seeded with tumor.

### Systemic deuterium labeling

The dMRI studies were performed per the experimental schema described in Fig. 2 either 7 or 14 days after tumor cell implantation. For these imaging studies, a ^2^H_2_O level of ~ 8% in total body water (TBW) was targeted. The mice initially received two bolus injections (35 ml/kg body weight) containing NaCl (0.9%, w/v) in ^2^H_2_O (100%, Cambridge Isotope Laboratories) 24 h apart, each bolus increasing the TBW enrichment by ~ 4%. Thereafter, the mice were provided drinking water containing 16% (v/v) ^2^H_2_O (70% ^2^H_2_O, Cambridge Isotope Laboratories, diluted with sterile ultra-pure water, Quality Biological) until imaging was performed^21^. Administration of 16% (v/v) ^2^H_2_O was necessary to maintain 8% ^2^H_2_O in TBW taking into account the 30–40% loss of ^2^H_2_O due to respiration and perspiration^21^. ^2^H_2_O dosing was either initiated the day prior to tumor cell injection or 6 days after as indicated in the figure legend. For the dose-escalation study, mice received 1–4 bolus injections within 7 days prior to tumor cell injection and were subsequently provided 8%, 16% and 32% ^2^H_2_O drinking water to achieve targeted concentrations of 4%, 8% and 16% ^2^H_2_O, respectively in TBW based on previous studies^21^. Regular drinking water served as a control (0.015% ^2^H_2_O).

### Urine/serum TBW deuterium enrichment measurement

Urine and blood sampling has been used interchangeably to quantify TBW ^2^H_2_O enrichment, as previously shown^36^. As mice recovered from anesthesia on a heating pad, ~ 50 μl of urine was collected on a sheet of parafilm upon spontaneous passage. The urine was immediately transferred to a plastic microcentrifuge tube. When serum was used, it was collected via mandibular or retroorbital bleed and allowed to clot for 30 min at room temperature. The samples were spun down at 2655×*g* (5417R, Eppendorf) and the supernatant was transferred to a new microcentrifuge tube. Urine and serum samples were stored at − 20 °C until TBW ^2^H_2_O enrichment analysis using head space gas chromatography-negative chemical ionization mass spectrometry (HS-GC-NCI-MS) was performed as previously published^36^.

### Tissue sample collection and preparation for GC–MS/MS enrichment analysis

Isotopological enrichment of deuterium in the DNA base deoxyadenosine (dA) was determined in tissue samples of HT-29 tumors and anterior thigh muscle of the contralateral hind limb. Tissues were excised immediately post-mortem after imaging and stored at − 80 °C. Subsequently, we used a modified version of our validated GC–MS/MS method for analysis^37^. Briefly, after isolating DNA from mouse tissue samples using a tissue DNA extraction kit (Maxwell® 16, Promega), the purified DNA was incubated and hydrolyzed enzymatically (EpiQuik, Epigentek Group Inc.) to its nucleoside bases (e.g., dA, dT). The method employed solid phase extraction (Waters HLB) to extract and purify dA (unlabeled and deuterium labeled) from leg muscle and tumor tissue, with automated on-line methylation (derivatization) and rapid chromatographic analysis (~ 6 min) using an Agilent GC–MS/MS system (7890A GC, LTM Series II Fast GC Module, 7000C GC–MS/MS Triple Quadrupole, 7693 Autosampler and 7697A Headspace Sampler, all Agilent Technologies). The prepared samples were injected into the GC using the following conditions, 1 µl pulsed split-less injection at 235 °C; component separation using low thermal mass DB-17MS column 15 m × 0.25 mm ID × 0.25 µm film with column oven temperature program from 50–320 °C at 120 °C/min. The MS used positive chemical ionization (PCI with isobutane reagent gas) and full scan mode (150 to 350 Da) to acquire MS data for evaluation.

As depicted in Supplementary Fig. 1B,C, MS overlays (normalized) of methylated dA and its isotopologues (e.g. dA M + 1, dA M + 2, dA M + 3 etc.) depict the stable isotopes of ^13^C, ^15^N, ^2^H, ^18^O found naturally (i.e. ~ 23% background) in methylated dA and its isotopologues (Supplementary Fig. 1B, contralateral leg muscle, control), as well as enrichment of ~ 27% deuterium (~ 50% minus natural isotopic background) into the DNA base dA of rapidly proliferating cells (Supplementary Fig. 1C, HT-29 tumor).

### Proton and deuterium magnetic resonance spectroscopic imaging

MRI experiments were performed on an 11.7 T (Magnex Scientific, Figs. 2, 3) or 9.4 T (Biospec 94/30; Fig. 4, Supplementary Fig. 3) MRI equipped with a Bruker Avance or Avance III MRI console (Bruker-Biospin) and a custom in-house built elliptical dual-resonance transmit/receive coil consisting of an inner elliptical solenoid deuterium coil and a saddle proton coil (Supplementary Fig. 2A–C). The mice were imaged with both legs perpendicular to the B0 field. The homogeneity of both coils was tested using a tight fitting 3-D printed customized oval bottle that contained two compartments with regular water and water enriched with 5% ^2^H_2_O (Supplementary Fig. 2D).

Mice were anesthetized with isoflurane (4% for induction and 1.5–2.5% for maintenance in medical air, 500 ml/min). During anesthesia, the respiratory rate was monitored with a pressure transducer (SA Instruments Inc.) and maintained at 60 ± 10 breaths per minute. Core body temperature was also monitored using a nonmagnetic rectal temperature probe (FISO) and maintained at 36 ± 1 °C using a circulating water-warming pad. Immediately following anesthesia, both hind limbs were placed into the ^1^H/^2^H coil. For dMRI, three 3 mm slices with 3.5 mm voxels were acquired by chemical shift imaging without ^1^H decoupling using standard linear k-space CSI pulse acquire sequence with a 397 ms repetition time, 30° flip angle, 512 FID points, and a sweep width of 4000 Hz and a Hermitian excitation pulse. No correction for B0 and B1 + inhomogeneities was applied. Due to difficulties in proper phase adjustment arising from the susceptibility artifact near the ^2^H_2_O glass tube in the HT-29 images (Figs. 2, 3), the spectra in each voxel were processed in magnitude mode. MiaPaCa-2 MRI images (Fig. 4) were therefore acquired without a phantom vial attached to the leg. The FIDs were then zero-filled in k-space to a final size of 128 × 128 × 3. One average was acquired for each scan for a total scan time of 27 min.

A noise reduction algorithm was employed in post-processing^38,39^. The low rank tensor decomposition technique takes advantage of the fact that any multidimensional array can be decomposed exactly into a weighted product of vectors^40^, in this case vectors representing spectra and vectors representing image columns and rows. The full product considering all vectors reconstructs the image exactly. Truncating the expansion constructs a simpler form of the image. The repeating structure of the data (the peaks are in nearly the same position in every voxels, although the relative intensity varies) ensures that the expansion can be truncated early while preserving high fidelity to the image, eliminating noise (which dominates the later terms) with little impact on resolution^38,39^. Each dataset consists of 3 slices of 128 × 128 images with each voxel containing a 512-point spectrum. This is reconstructed by tensor decomposition using a low rank representation using 32 out of the possible 128 vectors in the x and y directions, all 3 slice vectors, and 32 out of 512 vectors in the spectral dimension^39^. Using this method, 92 ± 4% of the variance was captured in each scan, with the residual approximately corresponding to the noise level by visual analysis. The water peak from ^2^H^1^HO was set to 4.6 ppm relative to the sodium trimethylsilylpropanesulfonate (DSS) chemical shift reference, following a temperature correction to 37 °C^41^. No signal was detected when ^2^H_2_O labeling was omitted. The anatomical imaging was performed using the following parameters: spin-echo sequence (RARE, rapid acquisition with refocused echoes), TR/TE = 3000 ms/12 ms, with the same geometry as chemical shift imaging but with a matrix size of 128 × 128.

To compare signal between tumor and non-tumor areas, a spectrally selective image was first formed by summing the 10 points (156 Hz) on either side of the ^2^H^1^HO peak after noise reduction. A region of interest was then drawn around the right and left leg for each of the three slices in ImageJ^42^, following the contours of the tumor on the anatomical RARE image for the Mia Paca-2 and encompassing the whole leg for HT29. The mean greyscale value was tabulated for each region and a ratio between the signal intensity of the tumor-injected and the normal leg was calculated for both spectrally selective images. Greyscale values were scaled to the intensity of the highest voxel in each image.

### Statistical analysis and software

For HS-GC–MS/MS operation, data acquisition and processing, we used a PC workstation with Agilent MassHunter Acquisition (B.07.06.2704), Quantitative (B.08.00) and Qualitative (B.07.00, Service Pack 1) software. Data were analyzed and visualized using GraphPad Prism 8.0. All graphs represent the mean value ± SEM. Statistical analysis was performed as indicated in the figure legends with p < 0.05 considered significant.

## Results

### ^2^H_2_O intake leads to dose-dependent labeling of deoxyadenosine (dA) in tumor cells

**Figure 1:**
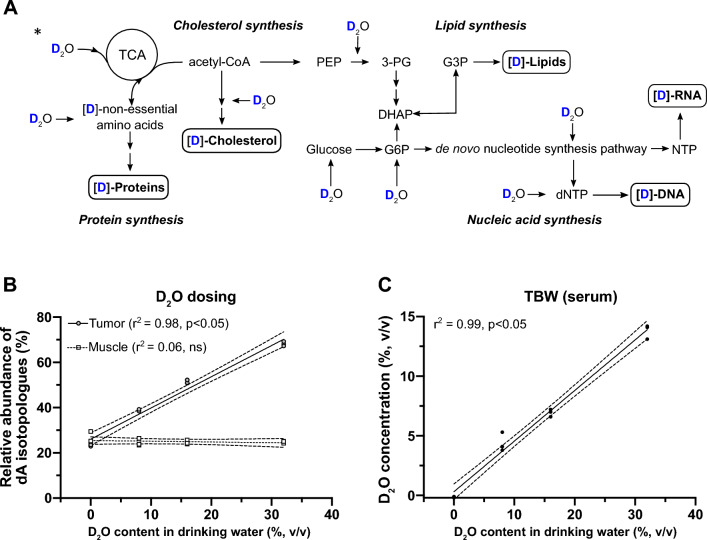
Deuterium is incorporated into biomolecules during systemic administration of ^2^H_2_O leading to a dose-dependent increase in the tumor isotopologue abundance of dA. (**A**) A diagram of intracellular biosynthetic pathways that use water as a substrate and are, therefore, potential routes for non-exchangeable, stable deuterium incorporation into carbon-deuterium bonds. (**B**) Dose-dependent increase of total dA isotopologue abundance (dA M + 1, M + 2, M + 3, M + 4, and M + 5) in HT-29 xenografts receiving a range of increasing concentrations of 2H2O in drinking water (0–32%, v/v). Two weeks after tumor seeding, tumors and muscle tissue from the contralateral hind limb (control) were excised, DNA was isolated and dA isotopologue abundance was quantified via GC–MS/MS. Statistical testing was performed using linear regression (n = 3 per group). (**C)** The ^2^H_2_O concentration in total body water was quantified using serum samples following a two-week in vivo labeling period with a range of increasing ^2^H_2_O concentrations (as in **B**). The quantification was performed via the established headspace GC–MS method and statistically tested using linear regression (n = 3 per group). *Of note, D_2_O can enter the TCA cycle directly by the reaction catalyzed by aconitase or fumarase. The reactions catalyzed by aconitase, however, results in a hydroxyl that is highly broadened by chemical exchange and is unlikely to be detected, while the reaction catalyzed by fumarase results in a non-exchangeable deuteron that may to be detected by magnetic resonance spectroscopy. *PEP* phosphoenolpyruvate, *3-PG* 3-phosphoglycerate, *DHAP* dihydroacetone-phosphate, *G6P* glucose-6-phosphate, *G3P* glycerol-3-phosphate, *(d)NTP* (deoxy)nucleoside triphosphate.

^2^H_2_O can replace water as a substrate in numerous biosynthetic pathways required for cell proliferation, Fig. 1A. When deuterated water is administered systemically, biomolecules within rapidly proliferating cells incorporate deuterium; hence, deuterium enrichment serves as a proxy for cell proliferation^20–22,43,44^. To estimate the degree of overall in vivo tumor biosynthetic labeling through systemic ^2^H_2_O administration we measured deoxyadenosine (dA) deuterium enrichment in purified tumor DNA using the gas chromatography tandem mass spectrometry (GC–MS/MS) method we developed^20,36,37^. Xenografted tumors were extracted following 7 and 14 days of in vivo growth and concurrent ^2^H_2_O labeling. The contralateral quadriceps muscle that was not injected with tumor cells served as a control for this analysis, Fig. 1B. In addition, we confirmed targeted total body water (TBW) deuterium enrichment on the day of imaging using our head space-gas chromatography method^36^, Fig. 1C.

dA was extracted and purified from the tumors that were excised from mice receiving a range of deuterated water concentrations in drinking water (v/v: 0%, 8%, 16% and 32%). Higher doses of ^2^H_2_O in drinking water led to an increased total body water (TBW) and tumor dA deuterium enrichment, which showed a linear dose-dependent pattern, Fig. 1B. In contrast, the enrichment of dA isolated from the muscle tissue (control) remained stable for the full range of tested TBW ^2^H_2_O concentrations (Fig. 1B), confirming that ^2^H_2_O preferentially labeled tumor cells. Additionally, we found that multiple deuterium atoms incorporated into the dA molecule, with more than half of the dA containing multiple deuterium atoms (e.g., dA M + 2, M + 3, Supplementary Fig. 1A).

### In vivo deuterium labeled HT-29 tumors were detectable by dMRI

#### dMRI distinguishes tumors from muscle tissue

**Figure 2:**
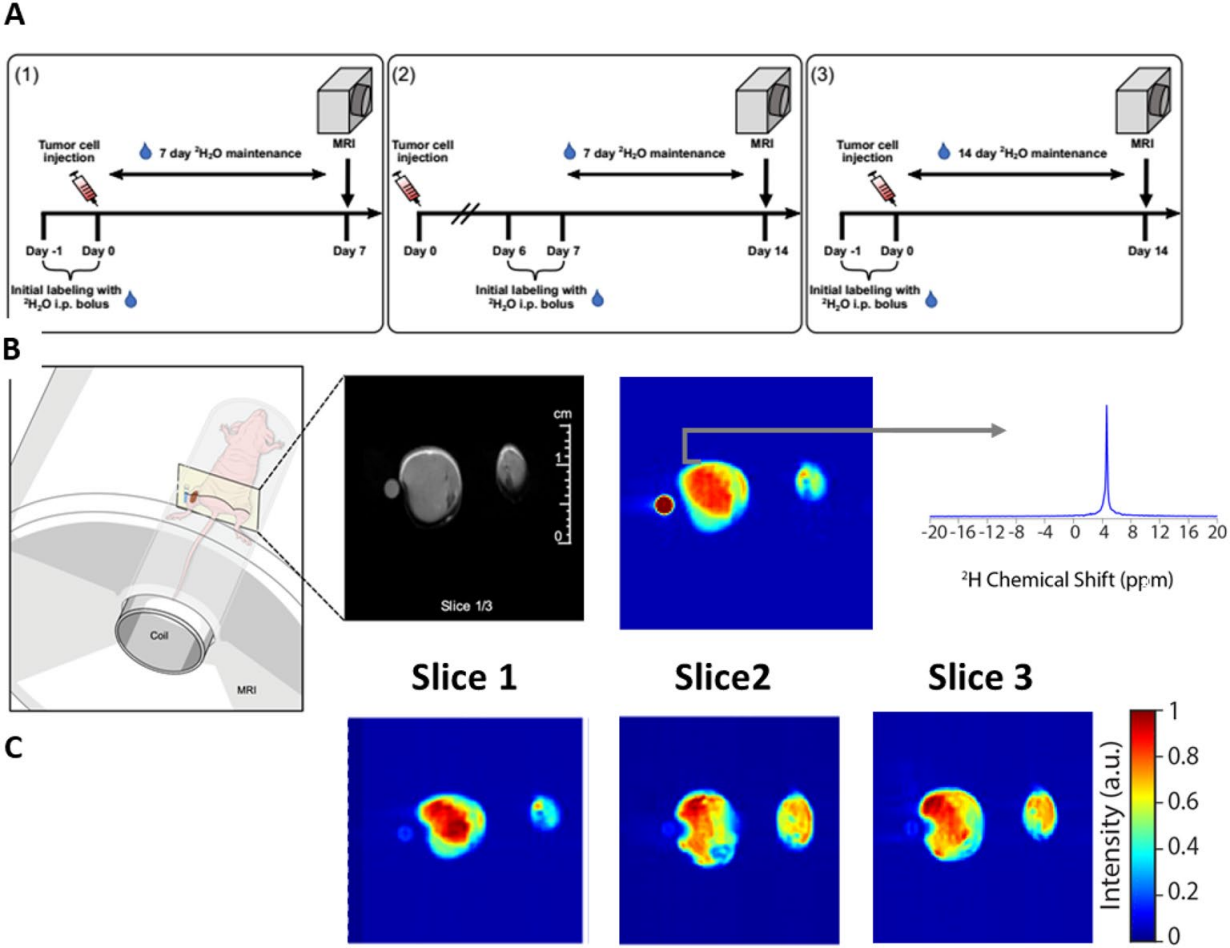
dMRI performed at 9.4 T on mice maintained at ~ 8% TBW and bearing HT-29 tumors in one hind limb demonstrates strong ^2^H^1^HO contrast within the tumor region. (**A**) Three labeling protocols were tested for imaging. Mice that underwent imaging were either labeled to ~ 8% ^2^H_2_O in TBW (v/v) by day 0 [(1) and (3)] or day 7 (2) and maintained at this TBW until imaging was performed, which was on day 7 and 14 after tumor implantation for (1) and (2,3), respectively. (**B**) Illustration of the imaging setup (left) with a representative deuterium image (right) along with the corresponding anatomical proton MRI image (center) using the labeling protocol described in (2), i.e., ^2^H_2_O labeling to 8% TBW on day 7–14 followed by dMRI on day 14. A phantom containing 10% ^2^H_2_O in H_2_O (v/v) was included with the scan for reference, as shown. (**C**) Spectrally selective images for the image in (**B**) using (**A**, (2)) labeling schema, corresponding to the integrated peak at 4.6 ppm (^2^H^1^HO chemical shift).

**Figure 3:**
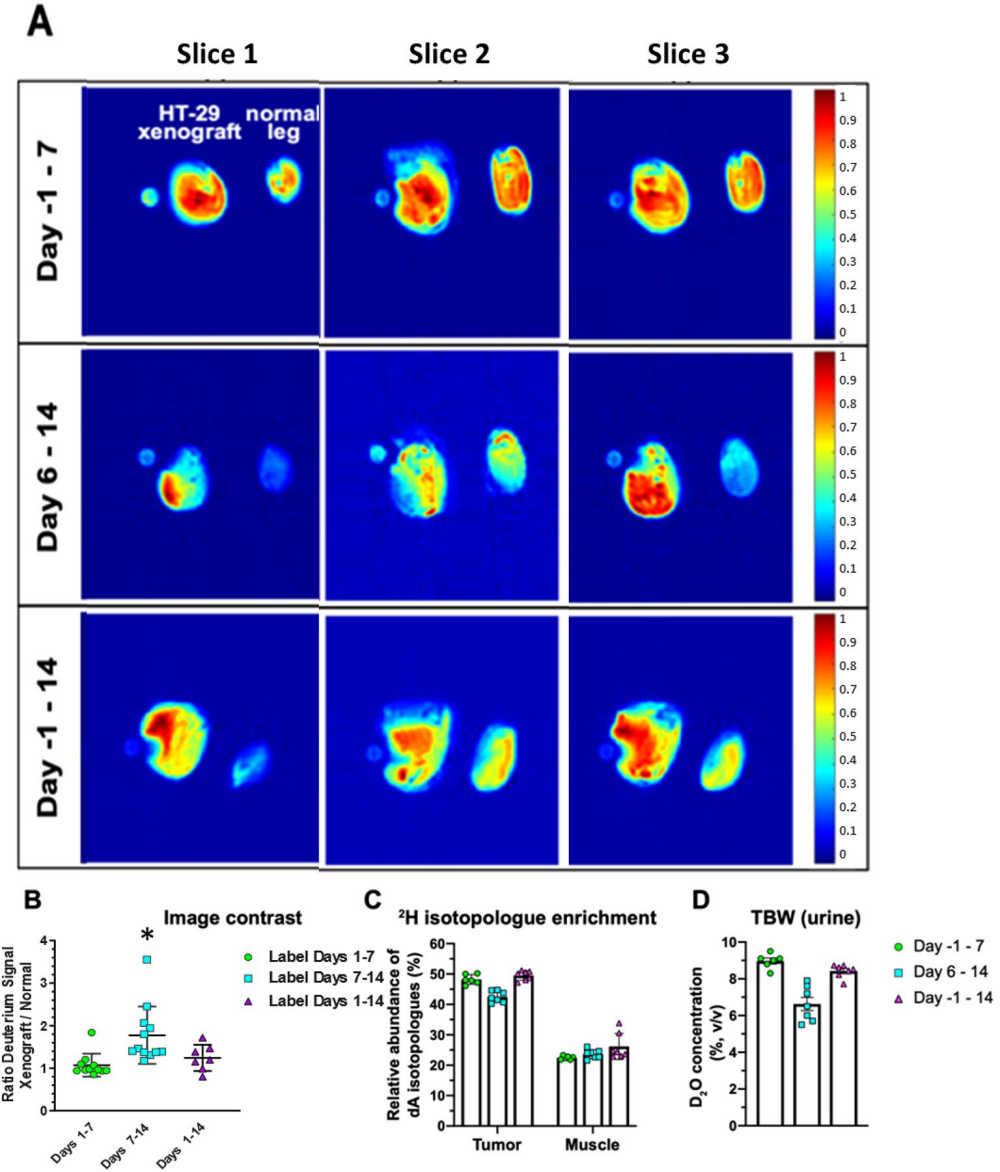
Comparison of different labeling schemas on HT-29 xenografts indicates that systemic ^2^H_2_O labeling from day 6 to 14 affords the highest dMRI contrast between the tumors and healthy muscle on day 14. (**A**) Representative images of the three labeling protocols for the 4.6 ppm (water) deuterium peaks across 3 slices of HT-29 tumors acquired on the 11.7 T scanner. (**B**) Quantification of the contrast between tumor-bearing and control hind limbs for the labeling schemas defined in Fig. 2A. The leg area was defined as a region of interest with the mean grey value measured within and expressed as a ratio between the HT-29-injected and normal leg. Statistical analysis was performed using a Wilcoxon signed rank test against a hypothetical median of one, *p < 0.05, n = 7 per group. (**C**) Relative abundance of dA isotopologues after a 7-day or 14-day labeling period in tumor and muscle tissue (n = 6–7 per group) analyzed via GC–MS. (**D**) The ^2^H_2_O concentration in total body water was quantified using HS-GC–MS on urine samples collected at the time of imaging.

We performed in vivo labeling on mice seeded with HT-29 tumors in a single hind limb and performed dMRI on both hind limbs as shown in Fig. 2, either 7 or 14 days after tumor seeding, Fig. 2A. We consistently observed a stronger ^2^H^1^HO deuterium signal at 4.6 ppm in the tumor region compared to healthy muscle (Fig. 3A). The strongest contrast was observed after 14 days of tumor growth with labeling starting on the 6th day (labeling schema (2) in Fig. 2A, cyan squares in Fig. 3B). Limiting both growth and labeling to the first 7 days (labeling schema (1) in Fig. 2A) led to negligible contrast. Labeling for the full 14-day tumor growth period (labeling schema (3) in Fig. 2A) was associated with a lower contrast, possibly due to increased background signal in the contralateral limb weakening the contrast.

### Selected labeling-imaging schema generates strong dMRI contrast in the pancreatic tumor model

**Figure 4:**
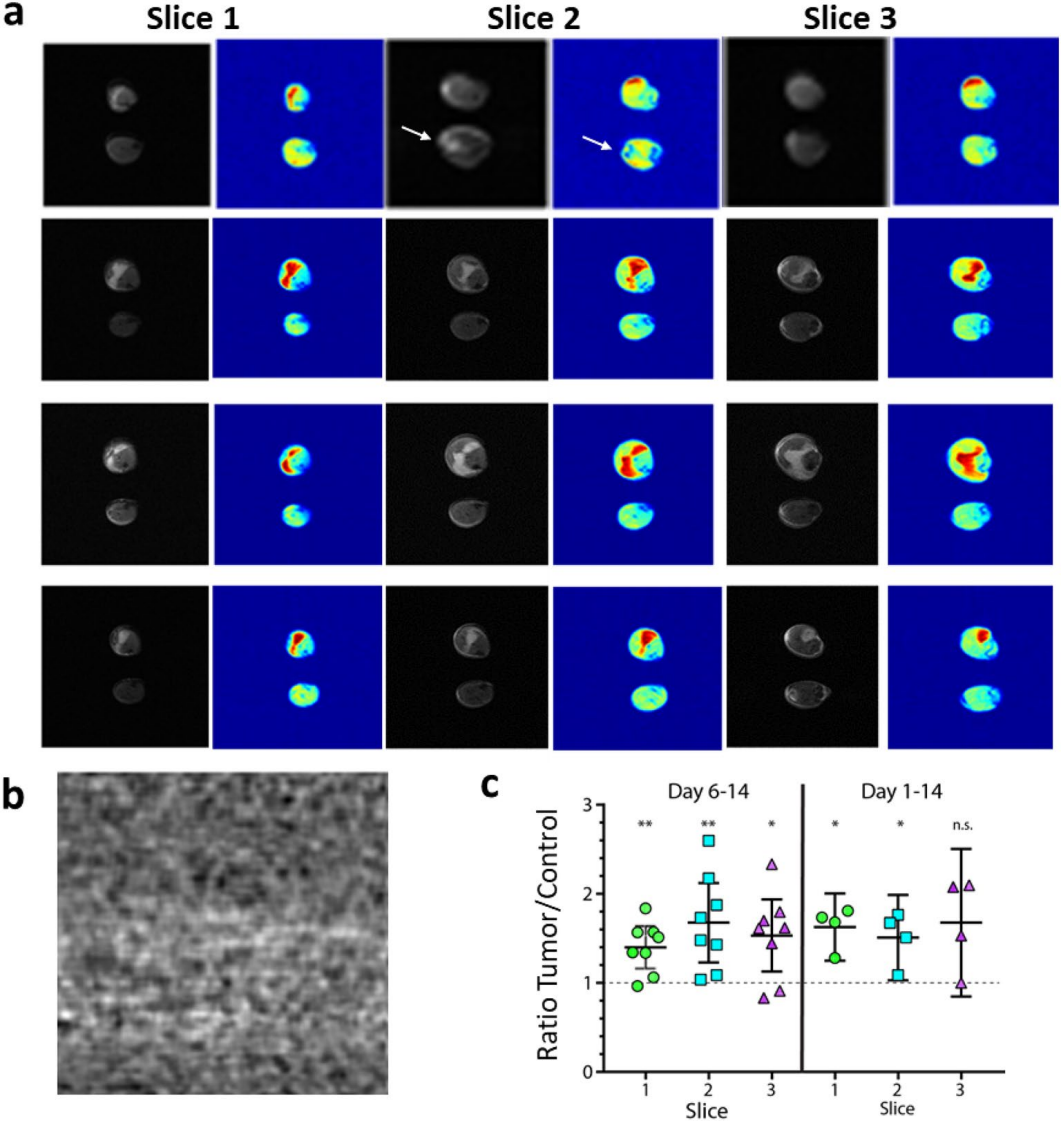
dMRI performed on MiaPaCa-2 xenografts labeled using the selected schema demonstrates similarly strong imaging contrast between tumor and healthy tissue at 9.4 T. (**a**) Representative paired RARE anatomical and deuterium CSI images at 4.6 ppm obtained on both limbs, MiaPaCa-2 xenograft bearing (top) and contralateral unmanipulated limb (bottom). Images were obtained 2 weeks after tumor seeding, with ^2^H_2_O labeling during week 2. White arrows indicate a tendon that appears hyperintense on the RARE but not the deuterium image. (**b**) dMRI signal in the in the absence of ^2^H_2_O labeling. (**c**) Quantification of the contrast between the tumor-bearing and control hind limb summed across the leg volume for the deuterium signal region for each of the 3 imaging slices acquired for each mouse. Statistical analysis was performed using a one sample test against a hypothetical median of one, n = 8, *p < 0.05, **p < 0.0.01. Error bars indicate the SEM.

To corroborate our findings, we tested the most promising ^2^H_2_O labeling strategy (d 6–14) in a second xenograft model using the pancreatic cancer cell line MiaPaCA-2 (Fig. 4). Tumor cells were subcutaneously injected into a single hind limb, and both hind limbs were imaged after 14 days of in vivo tumor growth. As previously demonstrated with the HT-29 model, deuterium labeling from day 6 to 14 provided a significant contrast, enabling a clear distinction between the tumor-injected leg and the contralateral limb (Fig. 4A,C). Compared to HT-29, MiaPaCa-2 tumor borders were more easily identifiable on the anatomical MRI, and the comparison of the deuterium signal with the anatomical image confirmed that the highest signal originated within the tumor tissue. Of note, some structures that appear hyperintense in the RARE MRI scans (tendon) did not show significant contrast in the deuterium image (Fig. 4A, white arrows).

## Discussion

In our study, xenografted tumors were labeled with deuterium in vivo during a proliferative period through the systemic administration of deuterated water. In contrast to proton density images, the deuterium contrast from ^2^H_2_O is a dynamic measurement that accounts for the uptake and elimination of the tracer. The advantages of a dynamic measurement have been shown in T cell activation, where H_2_^18^O tracer labeling showed an increase in metabolism derived water during the initial slow growth phase after T-cell activation and a rapid influx of extracellular water into the cytoplasm^34^, leading to a growth in cell volume independent of cell proliferation^45^. Systemic labeling with ^2^H_2_O in our study shows similar complex dynamics. The deuterium content in urine decreases in the second week of tumor growth (Fig. 3D). In this same time period, the tumor doubles in size^35^ while the deuterium enrichment of deoxyadenosine (dA) is essentially constant (Fig. 3C). These findings—an increase in deuterium ^2^H^1^HO contrast in the absence of an increase in the deuterium labeling of DNA—are consistent with an increase in cell size. dMRI may therefore find use as an approach to measure cellularity along with other indirect methods such as diffusion weighted imaging^46^. Most importantly, however, dMRI can provide strong imaging contrast between tumor tissue and surrounding healthy tissue. This lends rationale for further studies using deuterated isotopes for cancer imaging.

Although the current study was performed at high field, the overall SNR in our study was excellent (~ 150); hence, deuterium imaging at 3 T should be feasible^47^. Since short-term intake of low-to-moderate concentrations of deuterium is generally considered safe for humans and long-term toxicity studies in animals have not shown any detrimental effects below 20% TBW enrichment^24,25,48,49^, labeling cancer patients to 6–8% TBW may be safe and feasible. Nonetheless, since clinical studies involving deuterated water administration (for biosynthetic labeling) have so far only been performed at 1–2% TBW enrichment^50,51^, future studies would need to either lower the targeted TBW enrichment or incorporate close monitoring of patients during the labeling period to evaluate for possible adverse side effects. One logistical consideration for targeting a lower TBW enrichment is the volume of a single dose that can be given to a patient. According to Busch et al., intake of more than 70 ml of 70% ^2^H_2_O as a single dose can induce nausea and vertigo^21^. Hence, achieving 6–8% TBW enrichment with this oral/systemic labeling approach would take more than a week in the outpatient setting despite a similar TBW level being easily undertaken in study animals on the time frame of minutes. Given the strength of the observed dMRI signal in our preclinical study and the signal gain achieved by post-acquisition processing (~ 24-fold), future studies are planned to test lower TBW doses and shorter duration of systemic labeling, which would be advantageous for clinical translation of this approach. Additionally, while a CSI spectroscopic approach was used herein to verify the origin of the signal, acquisition can be accelerated by the use of a spin weighted imaging sequence once it is proven a single peak exists in the CSI imaging for the protocol being studied.

The first published use of deuterium in MRI was described in 1986^52^. It has since been used in animal models to visualize blood flow within a variety of tissues, including tumors^53–56^. In contrast to our approach, most studies used a single bolus injection of deuterated water to trace the deuterium signal in the organ of interest over a short time frame of minutes to hours. This enabled the visualization of blood flow and vasculature allowing the evaluation of tumor perfusion, but perfusion alone does not discriminate between healthy tissue and tumor. Continuous labeling with deuterium rather than administering a bolus of deuterium-labeled metabolite enabled us to enrich the tumor cells with ^2^H. Unlike deuterium imaging following a single bolus injection before image acquisition, the background signal of ^2^H_2_O in TBW in our protocol had equilibrated with the TBW; i.e. we were not evaluating a perfusion difference but rather the accumulation of ^2^H_2_O–^2^H^1^HO within the tumor. Additionally, we used dA deuterium enrichment to demonstrate and quantify the degree of nucleotide labeling, which estimates other biosynthetic labeling with deuterium^36,37^. Incorporation of deuterium into other major classes of biomolecules, such as amino acids/proteins^22,43^, triglycerides/lipids^57–59^, glycolysis^60^ and TCA cycle intermediates^61^, has been previously shown albeit through non-imaging approaches. Therefore, we infer that many biomolecules within the tumor are labeled with deuterium, and likely at multiple sites (see Supplementary Fig. 1b) given the highly anabolic nature of proliferating tumor cells. The biosynthetic labeling with deuterium during systemic administration of deuterated water may also be detectable by dMRI and subsequent studies to address this hypothesis are warranted; but at this time, the use of ^2^H_2_O deuterium imaging to detect other metabolites besides ^2^H^1^HO is speculative. Capturing downstream metabolites would require a different protocol, as the current setup is optimized to detect water uptake and retention differences in actively dividing cells over long periods. While the GCMS experiment establishes that other metabolites do get labelled by ^2^H_2_O it is unlikely that DNA or other macromolecules are detectable by MRI due their fast T2 relaxation.

Nonetheless, to elucidate this point, a different experimental design would need to be undertaken.

Currently, deuterated glucose is being developed as an alternative to 13C-pyruvate MRS given ease of clinical implementation and lower associated costs of the former^13,15^. DMI represents an excellent approach for elucidating tumor in vivo biology via short-term labeling and imaging of metabolism, but deuterated glucose is metabolized rapidly resulting in prompt loss of deuterium signal^62^. The spatial specificity for proliferating cells in combination with stable incorporation of deuterium and a robust SNR after image processing in our method provides an alternative for discriminating tumor tissue from surrounding healthy tissue. Additionally, our approach would be preferable in clinical settings where a large bolus of glucose is contraindicated, e.g., in diabetic patients or patients receiving corticosteroids. From an implementation standpoint, deuterium MRI is inexpensive with regard to label and system set-up, is technically straightforward, and can be used to evaluate multiple anatomical regions.

In summary, we have demonstrated that systemic administration of ^2^H_2_O leads to incorporation of deuterium into tumor cells in vivo. This enabled us to establish a dMRI approach that can distinguish a growing tumor from quiescent tissue in a non-invasive and non-radioactive manner. We believe that this proliferation-based labeling-imaging technique could provide an excellent alternative to existing cancer imaging approaches and allow non-invasive studies of cancer biology beyond metabolism.

### Supplementary Information

Supplementary Information.

## Data Availability

The data used and analyzed during the current study are available from the corresponding author upon reasonable request.
