# Supplementary material for: In vivo deuterium magnetic resonance imaging of xenografted tumors following systemic administration of deuterated water
Source: Sci Rep. 2023 Sep 7;13:14699. doi: 10.1038/s41598-023-41163-9 (PMC10485001; doi:10.1038/s41598-023-41163-9)
Supplement: Supplementary file 1 — Supplementary Information. [file 41598_2023_41163_MOESM1_ESM.docx]

**Supplementary Information**

**Figure S1:** Quantitative GC-MS analysis of deuterium enrichment into dA shows dose-dependent increase in the former with increasing TBW dosing and leads to multiple dA isotopologues, indicating that multiple sites on the dA molecule are enriched with deuterium. A) Representative dA isotopologue content (M+1, M+2,… M+5) within HT-29 xenografts labeled for 1 week at the stated deuterium concentrations in the drinking water, ranging from 0 to 32% (v/v). B) and C) Overlays of normalized MS scans (308, 309, 310, 311, 312, and 313 m/z) representing dA extracted and purified healthy muscle (B) and xenografted HT-29 tumor (C) from a mouse that was dosed to ~8% D_2_O in TBW (v/v) for 2 weeks. Summation of the relative abundances of the methylated dA component (retention time 4.74 min) for each dA isotopologue (e.g. dA M+1 (309 m/z), dA M+2 (310 m/z), etc.) in the muscle shows ~23% natural isotopic background (e.g. ^13^C, ^15^N, ^2^H, ^18^O) in the methylated dA component, while the tumor tissue shows ~50% dA enrichment, with ~27% ascribed to deuterium (~50% minus ~23% natural isotopic background resulting mostly from non-^2^H M+1 isotopes, e.g., ^13^C, ^15^N, etc.)

**
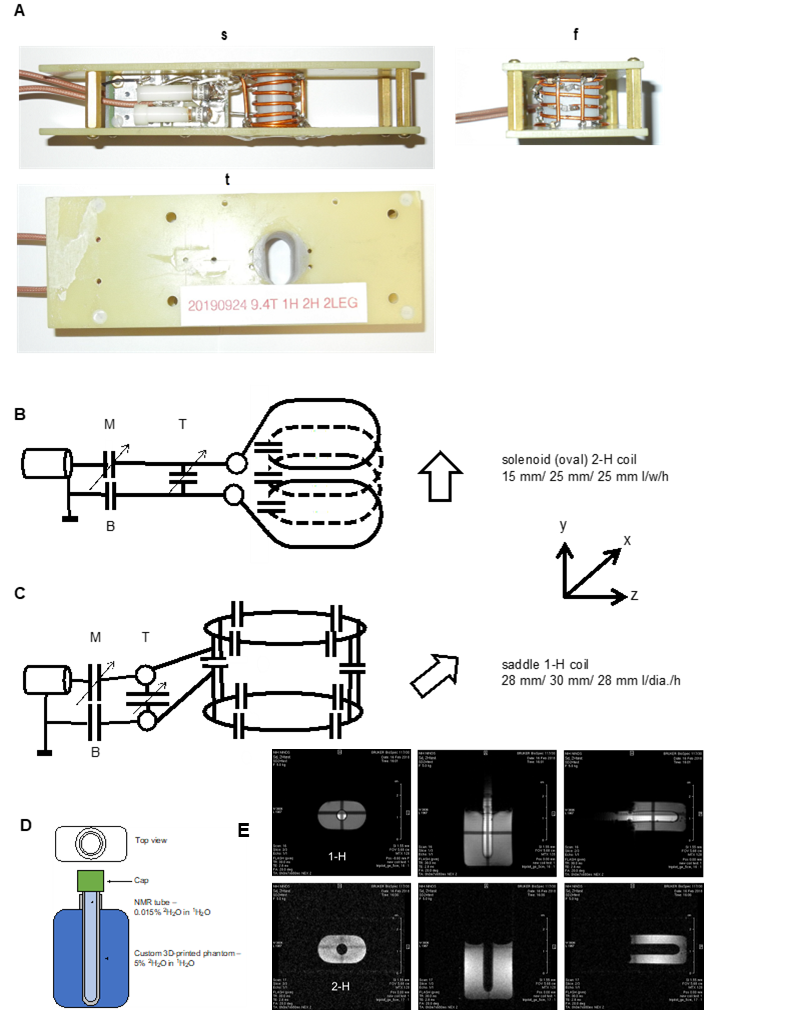
**

**Figure S2:** MRI coil and phantom schematics. A) The radiofrequency (RF) probes (coils) are embedded in a mechanical assembly made from two fiberglass boards that are separated by aluminum standoffs (s= side view; t= top view; f = frontal view). Photo Credit: Hellmut Merkle, Laboratory for Functional and Molecular Imaging, National Institute of Neurological Disorders and Stroke. B) An integrated oval shaped 3D printed hollow rod is the base for the windings of the 2-H oval-shaped solenoidal radiofrequency coil (height= 25.4 mm; outer short and long widths are 15 and 25 mm, respectively). The top board serves as a bed for the mouse and the inside of the hollow rod (33 mm long; 14 mm and 24 mm width) allows placement of its two hind legs. Radio frequency circuits and wire terminals for the coils are mounted on the bottom board. D) The 1-H saddle coil is in a ‘concentric’ shell placed around the 2-H coil but independently supported by top and bottom boards. Both RF probes are used in transmit/receive mode within the MR system. These are made from silver-plated 1.6 mm-thick varnished copper and integrated chip capacitors. Tune- and match-capacitors are variable high voltage tubular capacitors. The 2-H coil wires are tightly wound around the oval former and contain several in-line capacitors. The wires are attached to a standard tune/match circuit (M = match capacitor; T= tune capacitor; B = balance capacitor) that transform its impedance to the 50 Ω characteristic impedance of the RF transmission line. The 1-H coil has two saddle shaped branches that are also connected in parallel to a standard tune/match network. Both saddle branches have in-line capacitors. In addition, there are large decoupling capacitors inserted between branches to null their undesired interaction. The block arrows indicate the radiofrequency field direction B_1_ in coordinates where the main magnetic field B_0_ is in z-direction. D) Drawing of the custom-built phantom vial used to test image homogeneity and sensitivity. The phantom vial is filled with regular water (0.015% natural abundance of D_2_O) on the inside and 5% D_2_O in regular water (v/v) in the outer compartment. E) Test of the image homogeneity and sensitivity using the Bruker-TriPilot sequence at 9.4 T.

**
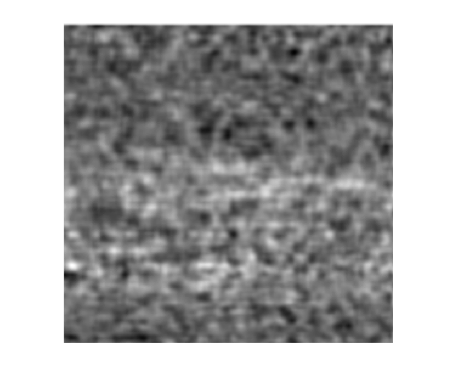
**

**Figure S3:** Example of a processed MR image taken from a mouse leg without labeling or tumor. The image was acquired using the same parameters and orientation as stated in Figure 4.
